# Supplementary material for: SurvCNN: A Discrete Time-to-Event Cancer Survival Estimation Framework Using Image Representations of Omics Data
Source: Cancers (Basel). 2021 Jun 22;13(13):3106. doi: 10.3390/cancers13133106 (PMC8269306; doi:10.3390/cancers13133106)
Supplement: Supplementary file 1 [file cancers-13-03106-s001.zip › Additional_File2.pdf]

SurvCNN: a discrete time-to-event cancer survival estimation framework using image representations of omics data (Supplementary tables and figures)

Yogesh Kalakoti[1*], Shashank Yadav[1*] and Durai Sundar[1#]

[1]DAILAB, Department of Biochemical Engineering & Biotechnology, Indian Institute of Technology (IIT) Delhi, New Delhi - 110 016, India;

*These authors contributed equally to this work

[#]Corresponding author

1 Supplementary tables

Table S1: Performance of the survival models for multiple combinations of omics datasets and different ways to represent the data. The total performance of 48 models is depicted using three metrics: C-index, Brier score, and IPCW score.

Table S2: Reference table for the twelve combinations of omics data analysed in the study. For every omics

**Omics type**	**Proportional Hazard (PH)**						**Non-proportional hazard (non-PH)**					
	TSNE			UMAP			TSNE			UMAP		
	C-Index	Brier	IPCW	C-Index	Brier	IPCW	C-Index	Brier	IPCW	C-Index	Brier	IPCW
**I**	0.683	0.152	0.695	0.690	0.172	0.695	0.689	0.147	0.701	0.692	0.173	0.713
**II**	0.672	0.154	0.664	0.680	0.193	0.675	0.679	0.130	0.672	0.692	0.173	0.713
**III**	0.551	0.183	0.575	0.669	0.193	0.666	0.679	0.130	0.672	0.673	0.177	0.662
**IV**	0.702*	0.168*	0.684*	0.694	0.199	0.651	0.524	0.732	0.517	0.643	0.167	0.647
**V**	0.674	0.161	0.706	0.651	0.168	0.642	0.673	0.138	0.717	0.668	0.151	0.655
**VI**	0.690	0.160	0.645	0.675	0.193	0.650	0.595	0.432	0.595	0.644	0.152	0.590
**VII**	0.690	0.190	0.686	0.688	0.183	0.713	0.691	0.161	0.702	0.686	0.171	0.694
**VIII**	0.677	0.188	0.667	0.684	0.189	0.680	0.681	0.139	0.675	0.683	0.141	0.697
**IX**	0.543	0.171	0.559	0.671	0.198	0.666	0.545	0.175	0.576	0.659	0.181	0.649
**X**	0.698*	0.165*	0.731*	0.702	0.187	0.658	0.529	0.582	0.526	0.651	0.175	0.663
**XI**	0.698	0.186	0.679	0.651	0.184	0.634	0.670	0.125	0.710	0.660	0.158	0.637
**XII**	0.679	0.155	0.660	0.673	0.187	0.647	0.589	0.439	0.546	0.632	0.163	0.622

** omics combinations denoted by roman numerals can be referred from **Table S2***

set denoted by roman numerals, '+' sign implies the inclusion of the corresponding omics data type.

	**I**	**II**	**III**	**IV**	**V**	**VI**	**VII**	**VIII**	**IX**	**X**	**XI**	**XII**
mRNA	+			+	+	+	+			+	+	+
meth		+		+		+		+		+		+
miRNA			+		+	+			+		+	+
Clinical							+	+	+	+	+	+

Table S3: Details of hyperparamers used for traing the ML model

Parameter	Value
Kernel Initializer	Glorot normal
Activation function	ReLU
Split ratio	0.2
Cross validation	10
Batch size	8
Learning rate	0.00001
Decay	1e-6
Momentum	0.9
Patience	25

2 Supplementary figures

Figure S1: Detailed methodology with CNN architecture for survival prediction model

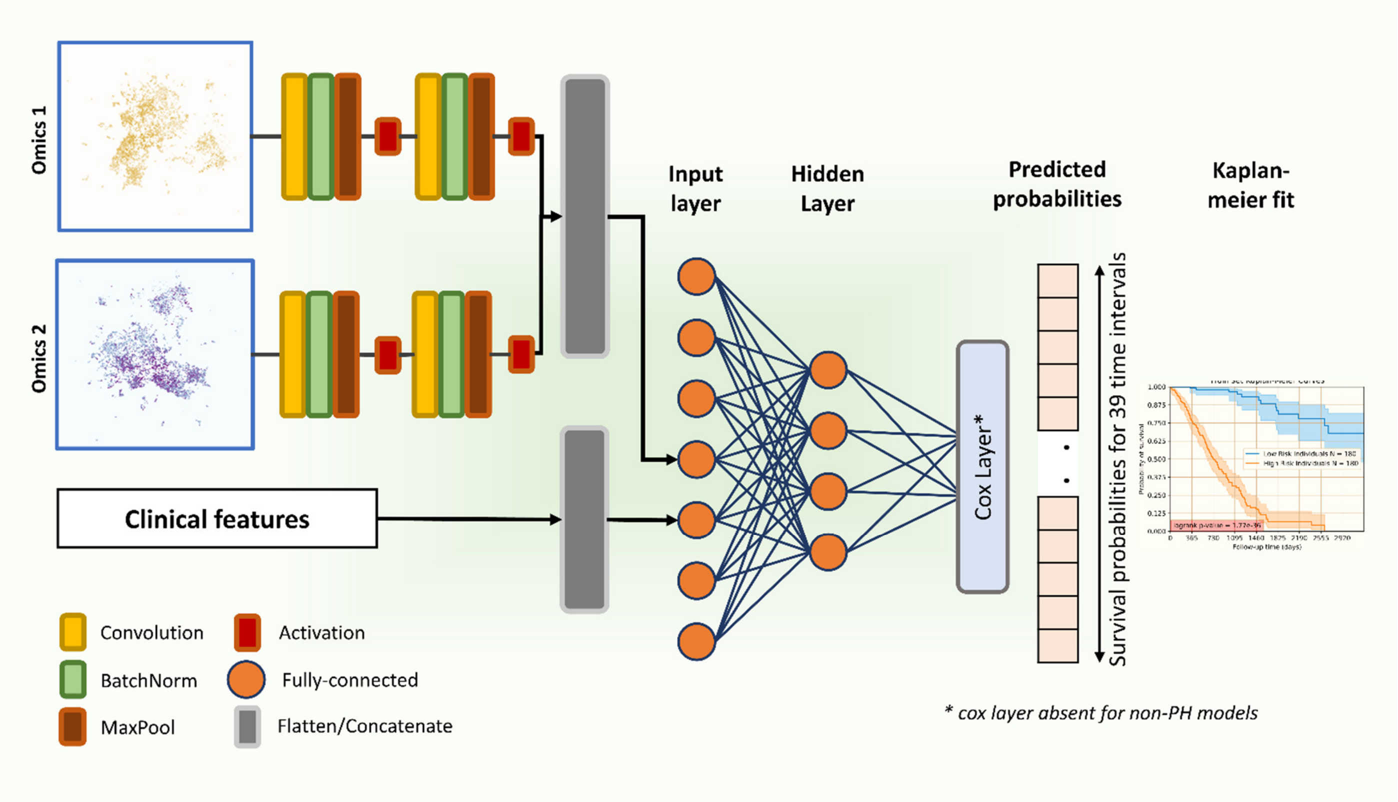

Figure S2: Model architecture for mRNA as an input (Omics combination type I)

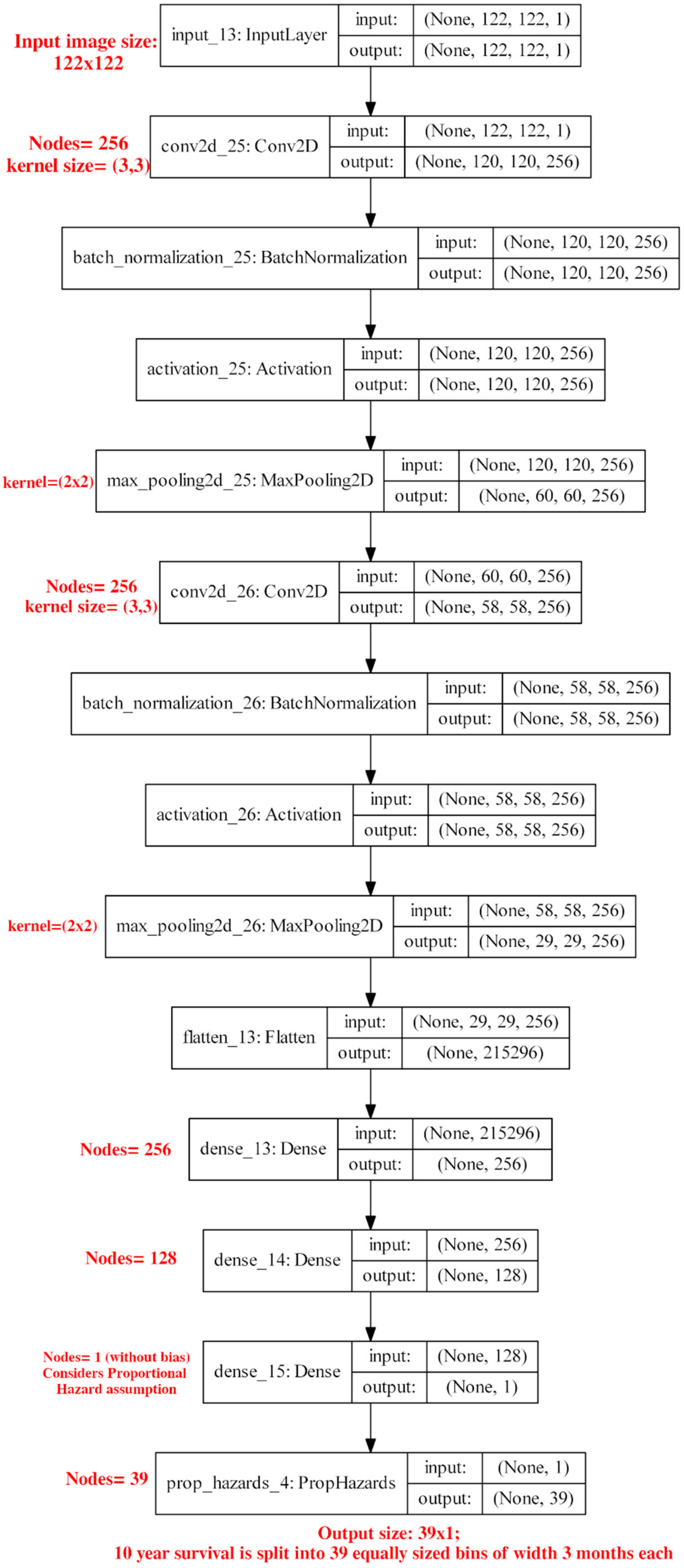

Figure S3: Model architecture for methylation as an input (Omic combination type II)

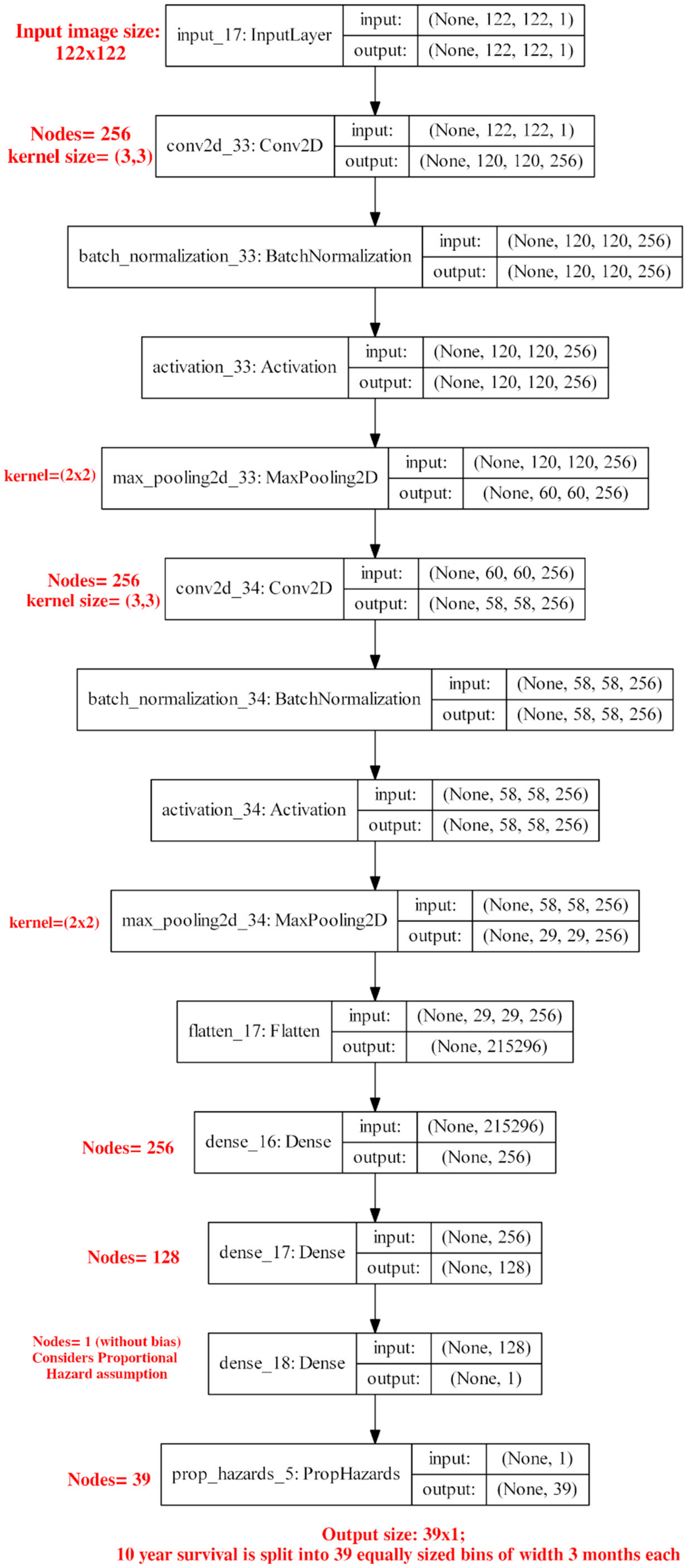

Figure S4: Model architecture for miRNA as an input (Omics combination type III)

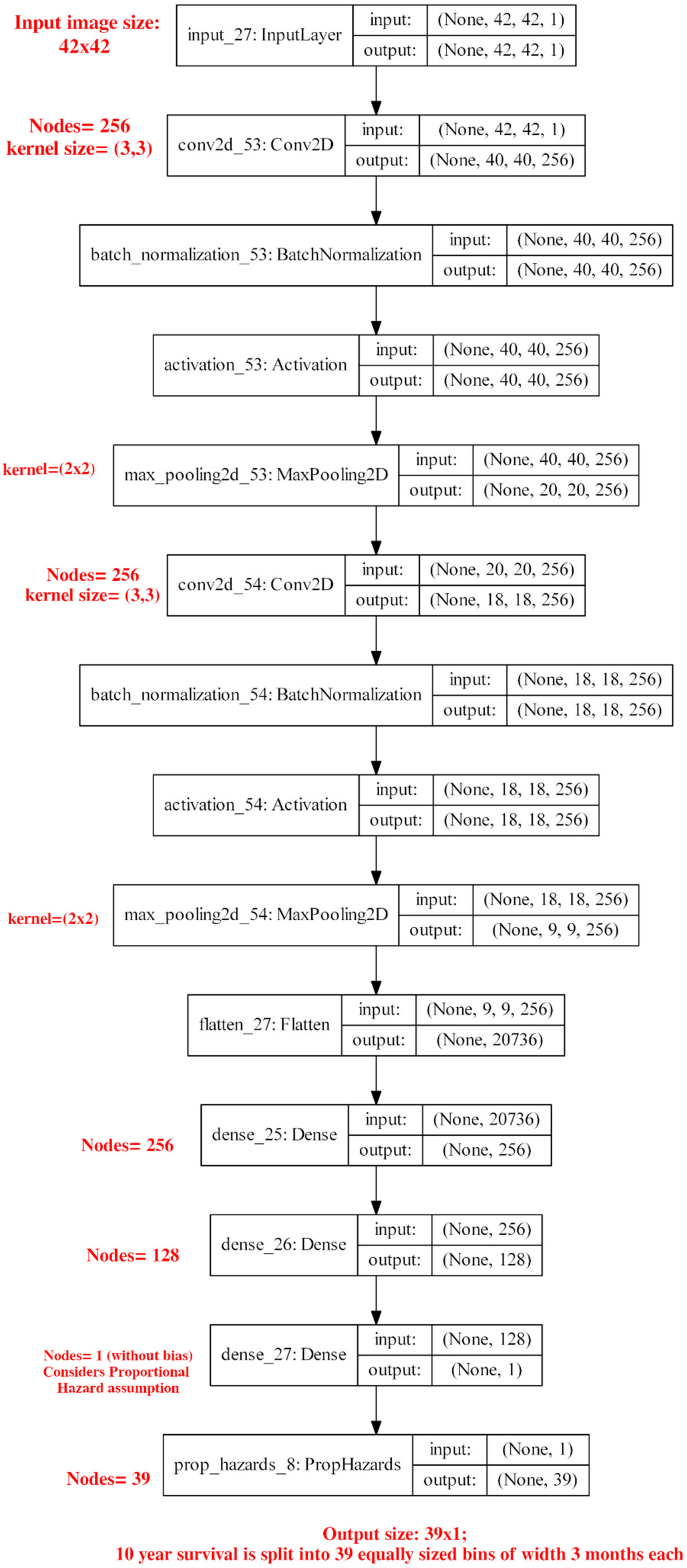

Figure S5: Model architecture for mRNA and methylation as inputs (omic combination type IV)

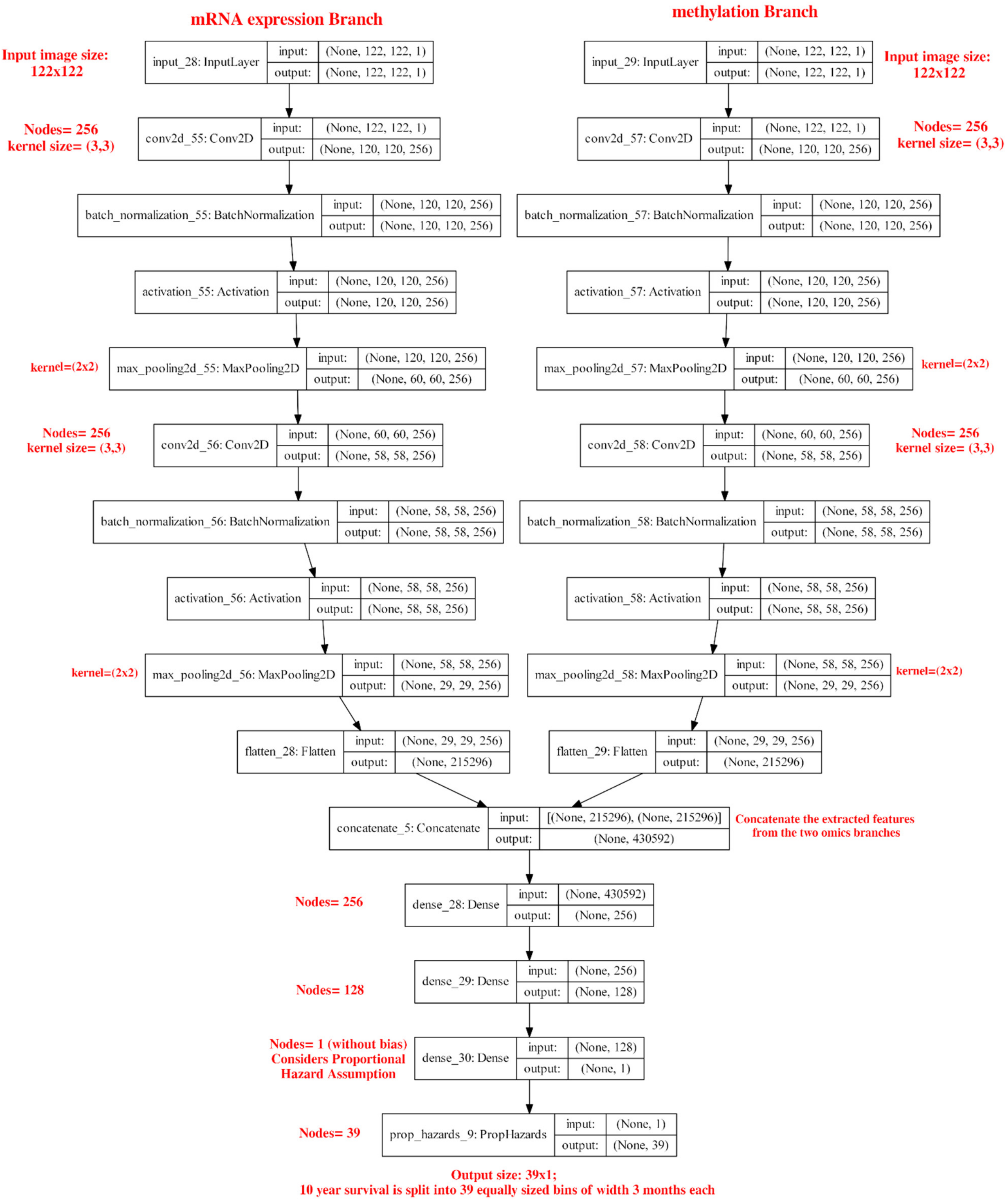

Figure S6: Model architecture for mRNA and miRNA as inputs (omic combination type V)

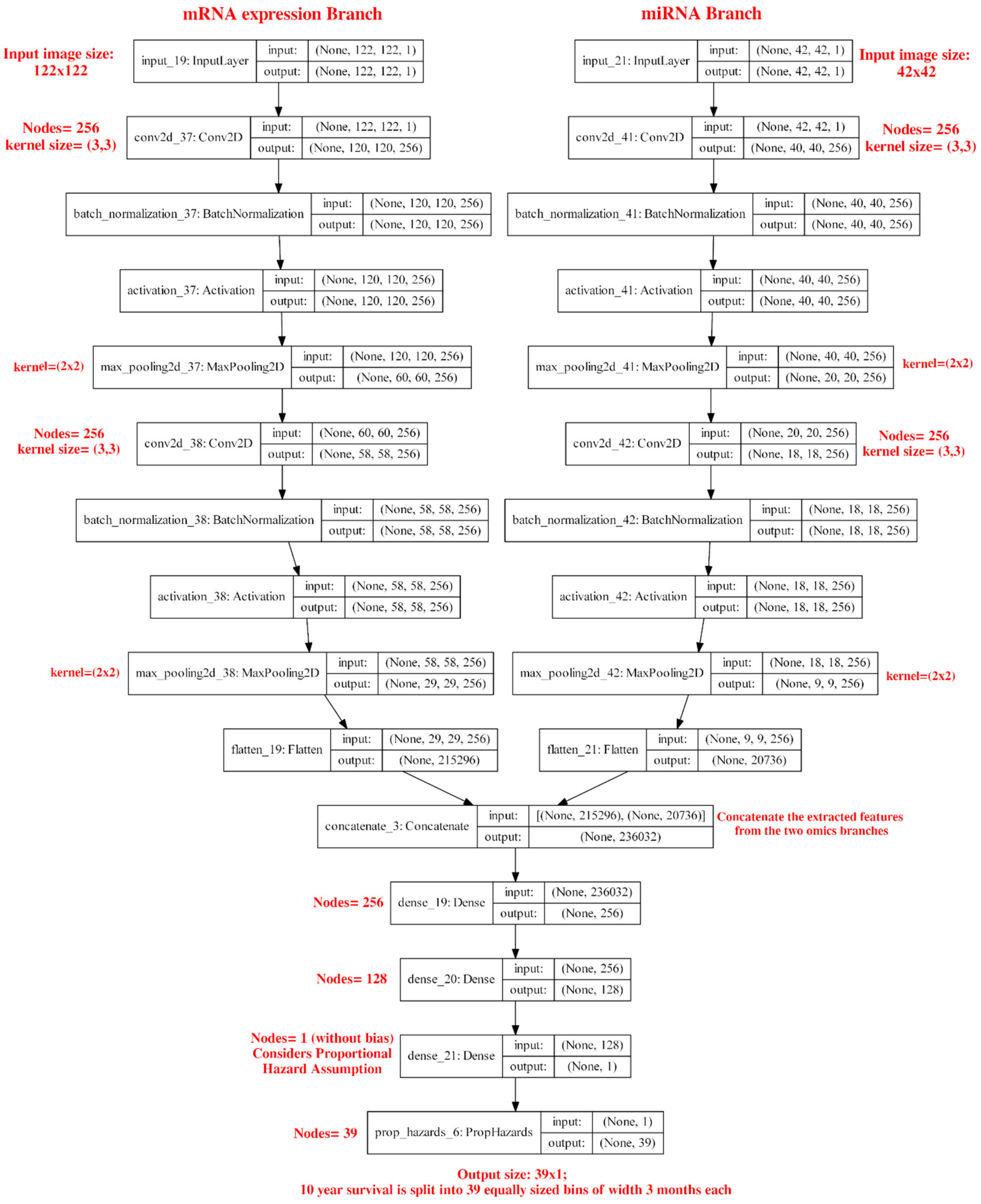

Figure S7: Model architecture for mRNA, miRNA, methylation as inputs (omics combination type VI)

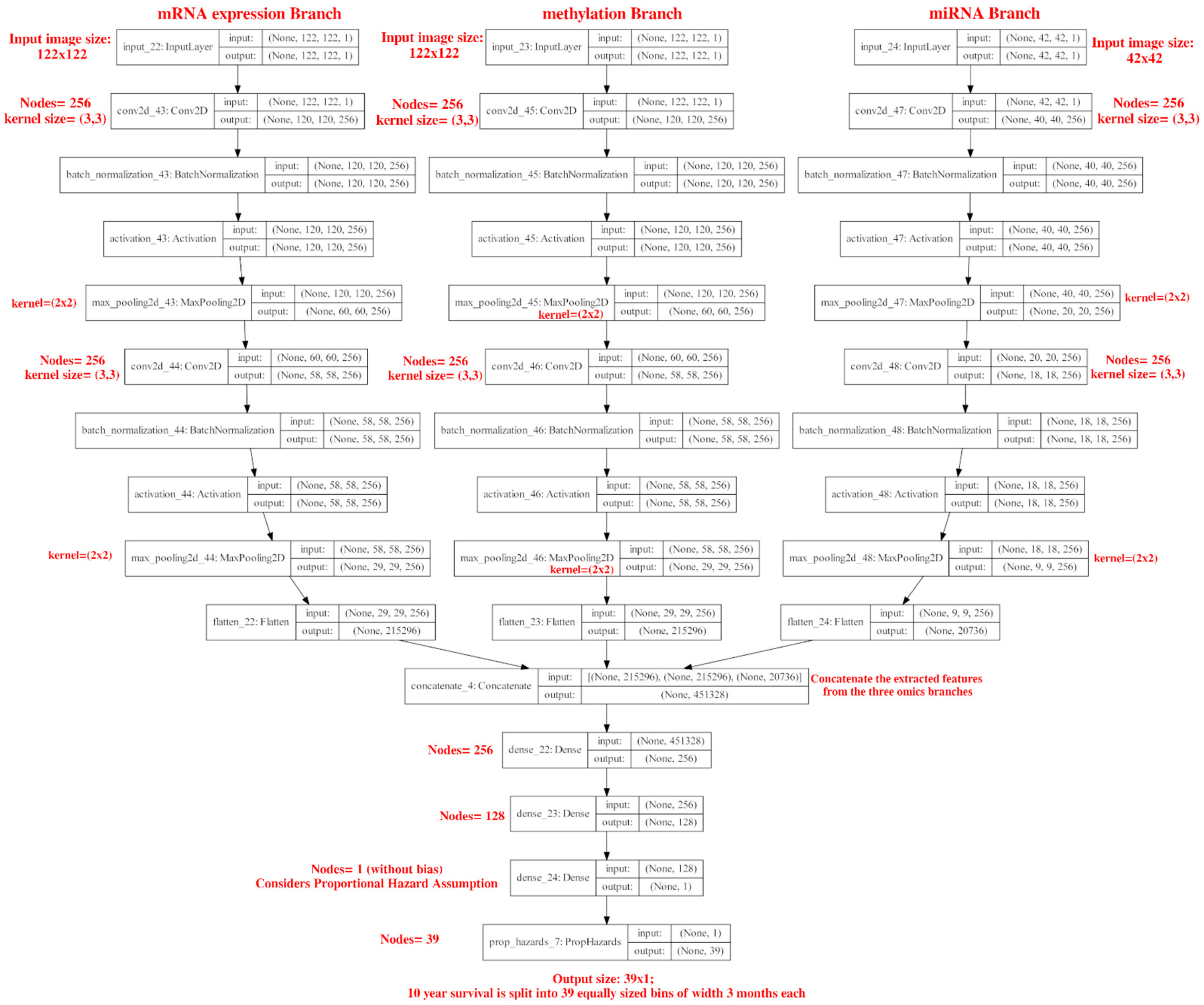

Figure S8: A stacked plot representing the number of patients that actually had an event that was predicted by the model. For the testing set, about 85% people in the low risk set were actually found to be living while, about 61% of high risk patients actually died during the given time period.

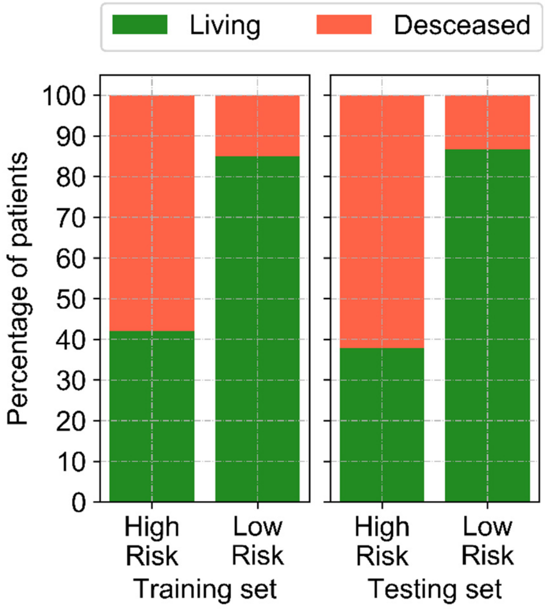

Figure S9: Enriched terms from KEGG 2019 Human pathways, GO Biological processes and Cell type (Human gene atlas) for (A, B,C) under-expressed gene-set.

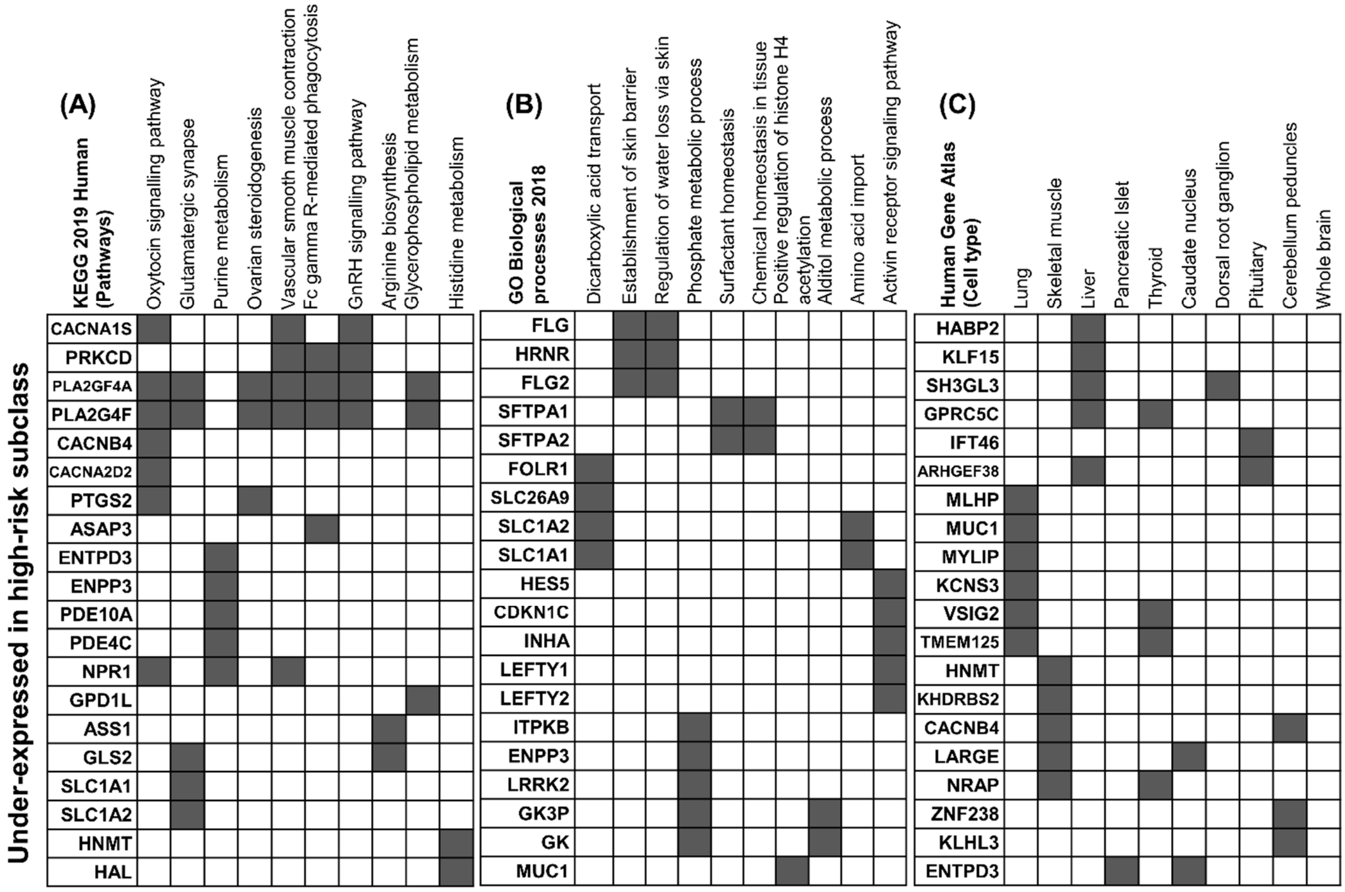

Figure S10: (A) Generate lower dimensional representations of data. (B) Find a minimum bounding rectangle and re-orient. (C) Label each point in the image according to the value in omics data For pixels with multiple genes, take an average and replace the value.

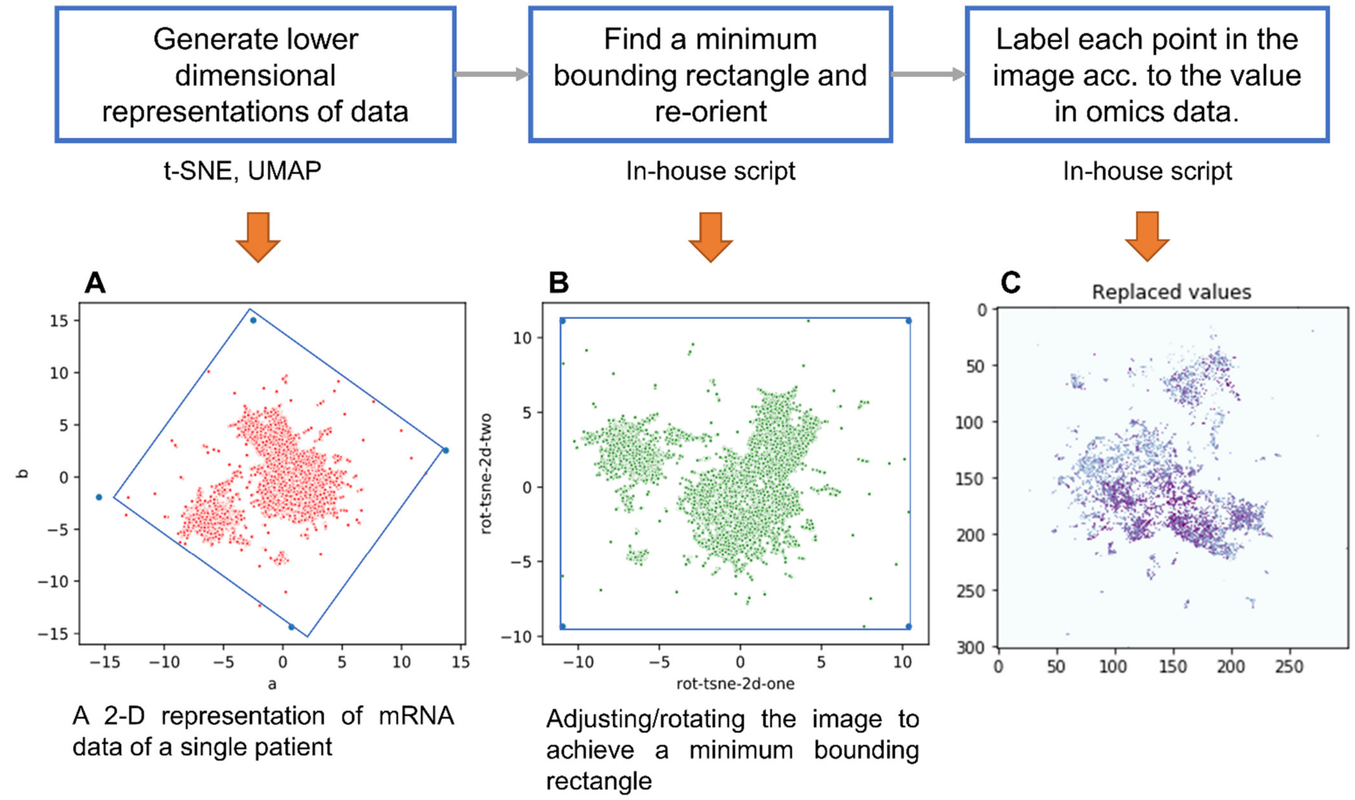

Figure S11: Silhouette analysis on the clustered cancer genes for both the approaches (t-SNE and UMAP) helped us to optimize the parameters used for the generation of images. For t-SNE, perplexity was optimized, while number of neighbours and minimum distance were optimized for UMAP.

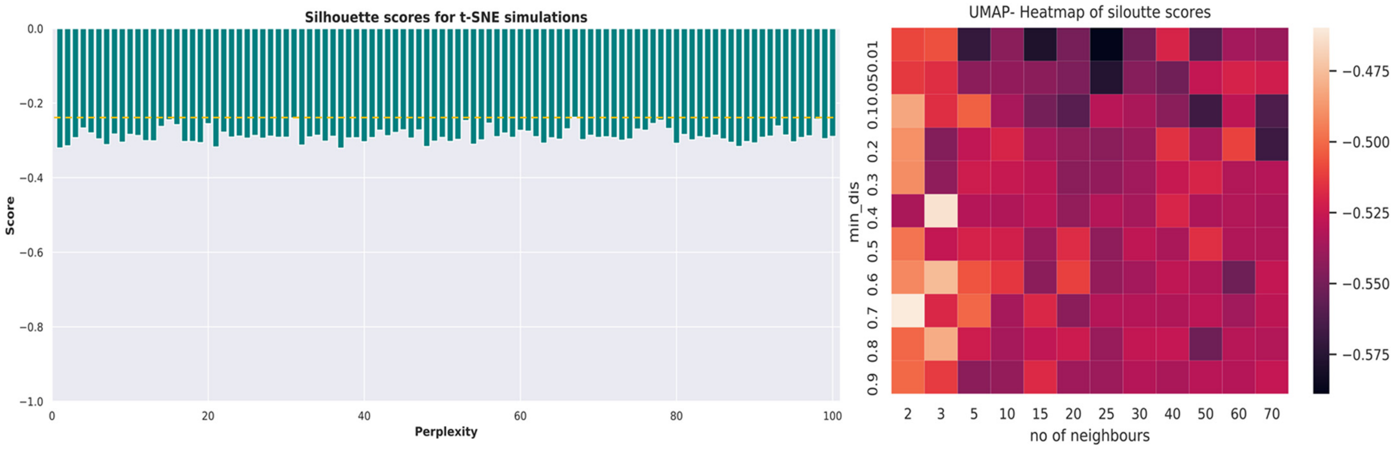

3 Supplementary notes (Feature transformation)

In this section, additional details of the feature transformation algoritm are summarised.

A general overview of the tranformation pipeline can can be found in Figure 1-B (manucript) and Figure S2. Let the numerical omics dataset consists of n samples and d attributes be defined as $\chi = \{x_1, x_2, x_3 \dots x_n\}$. Every element of χ is associated with a d-dimensional feature vector F which is defined as $\mathrm{F} = \{f_1, f_2, f_3 \dots f_d\}$. F is processed through t-SNE or UMAP to generate 2D coordinates $\{(a_1, b_1), (a_2, b_2), (a_3, b_3) \dots (a_d, b_d)\}$, where (a_i, b_i) represents the location of f_i , $i \in 1,2,3 \dots d$. The geenrated coordinates are graphically illustrated in Figure S4-A.

As we intend to use the image representaions on a Convolutional neural network, unnecessary white space had to be removed. Convex hull algorithm was used to find the mimumum bounding box for the coordinates as illustrated in Figure S2-B. The exact working and implementation of this algorithm can be found in the GitHub link provided for this study. Finally, the cartesian coordinates were converted into corresponding pixels in an image. Once the pixel coordinates are generated, the next step is to assign pixel intensities to this template for individual datapoints (patients). Therefore, for a set of n patients, n images will be generated.

Therefore, the entire process can be summarized in the following steps.

1. t-SNE/UMAP to generate the 2D mapping of the genes and saved the gene coordinates.
2. Calculate the minimum bounded rectangle covering all the gene coordinates using a Convex-Hull Finding Algorithm
3. Rotation and rescaling the cartesian coordinates to convert in to a square shape.
4. Convert the coordinates from cartesian dimension to pixel dimension.
5. For each patient map the corresponding gene expression (or other omics values) to pixel intensity.
